# STRIVE: interpretable multimodal subtype discovery in Women’s cancers using an attention-weighted joint variational autoencoder

**DOI:** 10.3389/fbinf.2026.1901464

**Published:** 2026-09-29

**Authors:** Zunaira Mehmood, Divya Sharma

**Affiliations:** 1 Department of Biostatistics, Dalla Lana School of Public Health, University of Toronto, Toronto, ON, Canada; 2 Department of Mathematics and Statistics at York University, North York, ON, Canada; 3 Department of Biostatistics, University Health Network, Toronto, ON, Canada

**Keywords:** artificial intelligence, cancer genomics, explainable AI, generative AI, multimodal AI

## Abstract

**Background:**

Breast, endometrial, and cervical cancers share a common challenge: molecular heterogeneity that single-modality profiling cannot fully resolve. Gene expression and DNA methylation capture distinct and partially non-overlapping regulatory programs, yet most subtyping frameworks treat them independently or merge them without accounting for modality-specific noise and scale.

**Objective:**

To develop an interpretable multimodal framework that integrates transcriptomic and epigenomic signals for principled molecular subtype discovery and molecular patient stratification with exploratory assessment of outcome associations across women’s cancers.

**Methods:**

We developed STRIVE (Stratification via Integrated Variational Embeddings), an attention-based joint variational autoencoder that learns modality-aware embeddings, fuses them into a shared latent space, and supports *post hoc* feature attribution. Subtypes were defined by K-means clustering on latent embeddings, cluster-defining signatures were derived using standardized mean differences (q < 0.05), and within-subtype survival prediction was assessed via permutation feature importance across multiple classifiers.

**Results:**

In TCGA-BRCA (n = 866), STRIVE identified six molecular subtypes with coherent multimodal signatures, with DNA-methylation features prominently represented in specific cluster signatures. Within-subtype survival-status prediction showed variable performance across classifiers and subtypes. Replication in TCGA-UCEC (n = 454) and TCGA-CESC (n = 296) recapitulated key signature patterns, with DNA-methylation features prominently represented in specific cluster signatures and more consistent predictive performance in UCEC than CESC.

**Conclusion:**

STRIVE demonstrates that attention-weighted multimodal integration surfaces molecularly coherent subtypes in which cluster-defining signatures frequently included epigenomic features, a structure that single-modality approaches would obscure. These findings position STRIVE as a framework for multimodal molecular stratification and future evaluation of subtype-specific clinical associations.

## 1 Introduction

Cancers that disproportionately affect women, including breast cancer (BRCA), endometrial cancer (UCEC), and cervical cancer (CESC), are characterized by substantial inter-patient heterogeneity in tumor biology, clinical course, and treatment response, yet this heterogeneity remains incompletely resolved by current molecular subtyping approaches. Large-scale resources such as The Cancer Genome Atlas (TCGA) have enabled systematic profiling across molecular layers, creating an opportunity to connect genomic and epigenomic variation to clinically meaningful phenotypes. Translating these high-dimensional measurements into robust, interpretable patient subtypes that inform prognosis and treatment, however, remains an ongoing challenge.

Multi-omics integration offers a principled path toward more complete subtype characterization, but existing approaches face persistent limitations. Classical dimensionality reduction methods including PCA, ICA, and NMF yield interpretable representations for single-modality data but do not naturally handle the differences in noise, scale, and sparsity that arise when transcriptomic and epigenomic layers are combined (Engreitz et al., 2010; Fehrmann et al., 2015; Gaujoux and Seoighe, 2013; Kong et al., 2008; Liu et al., 2020; Shen-Orr and Renaud, 2013; Way et al., 2020). Nonlinear embedding methods such as t-SNE, UMAP, PaCMAP, and TRIMAP improve visualization of complex phenotypes but are sensitive to hyperparameters and are not designed for principled multimodal fusion (Kwon et al., 2025). VAEs provide a probabilistic framework for learning shared latent structure across omics layers and have been applied broadly in multi-omics integration and single-cell modeling (Bakhtiari et al., 2025; Bernstein et al., 2020; Boob et al., 2025; Cao et al., 2022; Cao et al., 2024; Choi et al., 2023; Cuomo et al., 2021; Gong et al., 2023; Heinen et al., 2022; Jha et al., 2020; Karlberg et al., 2024; Kshirsagar et al., 2022; Linder et al., 2020; Maizels et al., 2024; Pan et al., 2022; Rockel et al., 2025; Sadria and Layton, 2025; Wang et al., 2023; Wang et al., 2024; Xu et al., 2023; Yin et al., 2025; Zheng et al., 2022; Zhou et al., 2025). Recent extensions including multimodal hierarchical frameworks and cross-modality translation approaches such as Cobolt (Gong et al., 2021), scButterfly (Cao et al., 2024), BindVAE (Kshirsagar et al., 2022), ScDALI (Heinen et al., 2022), FedscGen (Bakhtiari et al., 2025), and VelvetVAE (Maizels et al., 2024) have further improved integration fidelity and extended VAE-based paradigms to new settings. Hybrid strategies including SyntheVAEiser (Karlberg et al., 2024) and diffusion-enhanced VAE frameworks (Choi et al., 2023; Sadria and Layton, 2025) suggest that careful architectural choices can improve both utility and interpretability. Despite this progress, most approaches either pool modality signals without accounting for their differential informativeness or produce latent representations that are difficult to link back to specific molecular features. Although several of these approaches were developed in single-cell settings, we draw on their general principles of latent-variable modeling, modality-specific representation learning, and multimodal fusion rather than on single-cell-specific assumptions such as technical dropout or cell-level heterogeneity. STRIVE applies these principles to bulk tumor profiles, where the central challenges are high dimensionality and differences in feature scale and dimensionality across modalities. DNA methylation was selected because it provides regulatory information complementary to transcript abundance and is available at sufficient subject-matched scale across the three TCGA cohorts.

These gaps define two persistent barriers to clinical translation. First, integrating modalities without explicitly modeling their distinct noise characteristics and scales can produce unstable subgroup definitions, particularly when biological signal is unevenly distributed across molecular layers. Second, unsupervised clusters are rarely validated against clinically meaningful endpoints in a systematic way, making it difficult to assess whether discovered subtypes carry genuine prognostic value or reflect technical variation. Together these limitations motivate approaches that handle modality-specific structure explicitly, provide interpretable links between latent clusters and molecular features, and evaluate clinical relevance directly.

To address these needs, we developed STRIVE (Stratification via Integrated Variational Embeddings), an attention-based joint VAE that learns modality-aware embeddings before fusing them into a shared latent space, using feature-wise gating to emphasize informative dimensions within each modality prior to fusion. The attention mechanism directly addresses the first gap allowing informative features within each modality to be adaptively emphasized rather than pooling signals indiscriminately. Post hoc feature attribution via permutation importance and standardized mean difference signatures addresses the second gap by linking latent cluster separation to specific molecular features and exploring their association with survival outcomes. We apply STRIVE to TCGA BRCA, UCEC, and CESC cohorts to identify data-driven molecular subtypes and explore their outcome associations. Collectively, this manuscript makes three key contributions: (i) an attention-weighted joint VAE for principled integration of transcriptomic and epigenomic data, (ii) an interpretable stratification pipeline that identifies cluster-defining molecular signals, and (iii) an exploratory outcome-linked evaluation framework for characterizing survival-associated features within the identified subtypes.

## 2 Methods

### 2.1 Study design

We used high-dimensional genomic data from The Cancer Genome Atlas (TCGA), focusing on cancers that predominantly occur in women: breast cancer (TCGA-BRCA), endometrial cancer (TCGA-UCEC), and cervical cancer (TCGA-CESC) (Goldman et al., 2020). Gene-expression and DNA-methylation data for all three cohorts were obtained from the same TCGA/GDC harmonized pipeline, ensuring that normalization was applied consistently across datasets. Gene-expression values (FPKM) were normalized by gene length and the number of reads mapped to protein-coding genes, following the standard GDC mRNA quantification workflow; and DNA-methylation beta-values were derived from array intensities as β = M/(M+U), where M and U denote the methylated and unmethylated probe intensities, respectively (Zhang et al., 2021). BRCA, UCEC, and CESC were modeled independently and were not pooled for model fitting or clustering; therefore, no cross-cohort batch-correction procedure was applied. In BRCA, tissue source site was additionally evaluated as a proxy for source-related technical variation and was not significantly associated with cluster membership (p = 0.363). Samples were included if matched gene expression, DNA methylation, survival, and phenotype data were available, ensuring completeness across modalities. The analytic cohorts comprised 866 BRCA samples, 454 UCEC samples, and 296 CESC samples. Within each cohort, features with low variation were removed to reduce noise and computational burden, and outliers were identified using a z-score criterion. After preprocessing, the retained feature counts were: 44,341 gene expression and 300,402 DNA methylation features for BRCA; 45,495 gene expression and 318,546 DNA methylation features for UCEC; and 43,182 gene expression and 311,579 DNA methylation features for CESC.

### 2.2 STRIVE: Stratification via integrated variational embeddings (attention-based joint VAE framework)

We developed STRIVE (Stratification via Integrated Variational Embeddings), an attention-based joint variational autoencoder (VAE) for multimodal representation learning and molecular patient stratification (Figure 1). STRIVE’s network architecture features an encoder composed of three core elements—two independent attention blocks for modality-specific feature processing and one downstream joint VAE encoder for latent distribution parameterization—coupled with two independent, parallel decoders tasked with modality-specific reconstruction. The rationale for this joint, integrative approach is supported empirically: independent clustering of gene expression and DNA methylation yields poorly concordant patient groupings (Supplementary Note S1), indicating that the two modalities carry complementary rather than redundant structure and motivating their integration within a single shared latent space.

**FIGURE 1:**
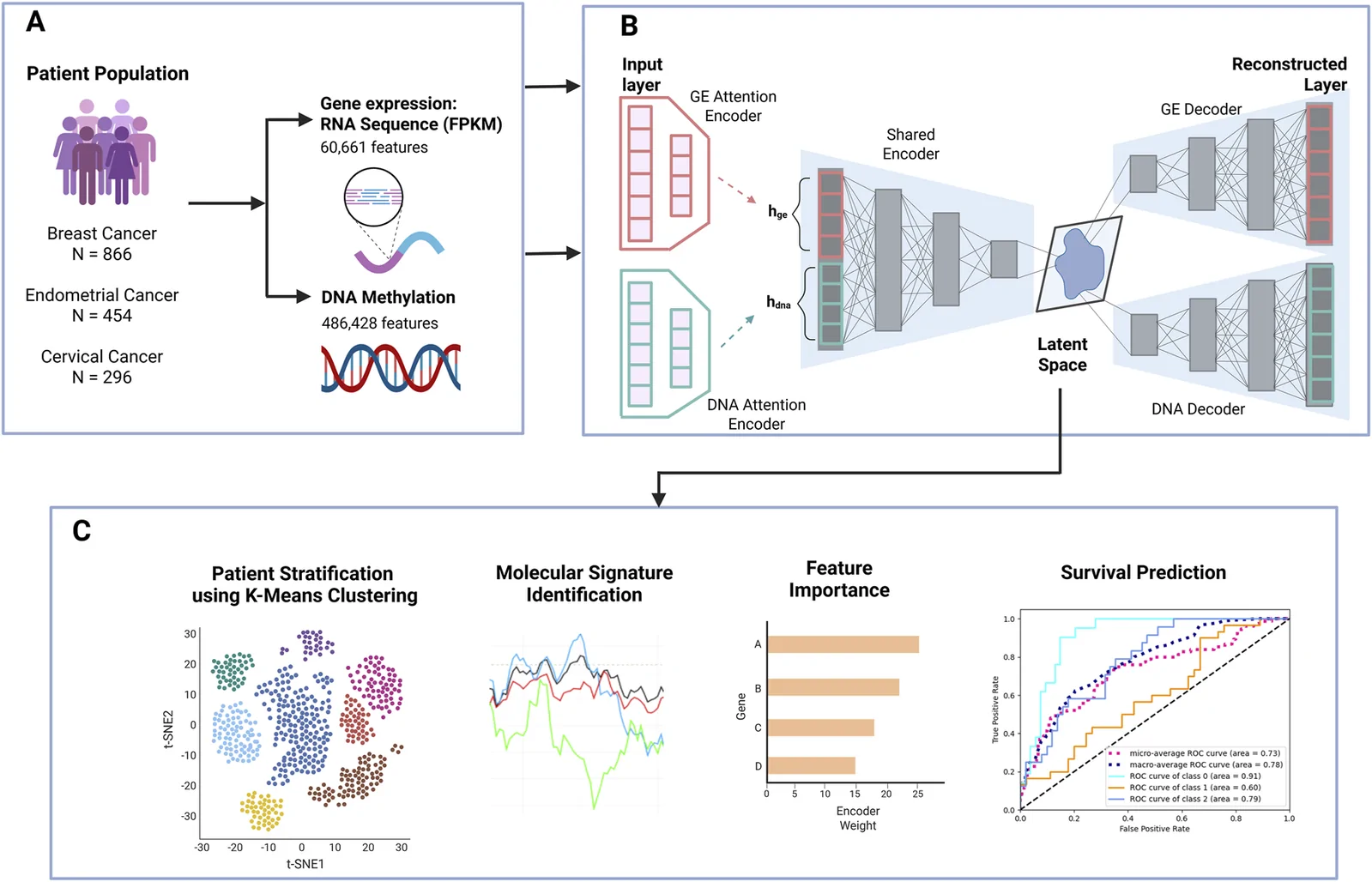
Overall framework of STRIVE for multimodal representation learning. STRIVE is an attention-based joint variational autoencoder (VAE) that integrates multi-omics data (gene expression and DNA methylation). Modality-specific attention encoders extract informative embeddings, which are concatenated and mapped to a shared latent space through a joint VAE encoder. Samples from this latent distribution are decoded through modality-specific decoders to reconstruct inputs, and latent embeddings are clustered using K-means. Following cluster identification, we applied three analyses to uncover molecular drivers and survival predictors: (1) encoder weight–based feature contributions, (2) cluster-specific gene signature identification, and (3) permutation feature importance for survival relevance. Created with BioRender.com. **(A)** Input. **(B)** STRIVE deep learning framework. **(C)** Outputs.

#### 2.2.1 Modality-specific attention blocks

Let $x=\left\{x_{GE},x_{{DNA}_{meth}}\right\}$ denote the raw multimodal inputs. Within STRIVE, each modality-specific encoder utilizes a sample-specific feature-wise gating mechanism designed to extract and prioritize feature-level importance localized within that specific modality. This intra-modality architecture operates independently on each data stream without calculating cross attention.

For each modality $m\in \left\{GE,{DNA}_{meth}\right\}$ , the network uses a dense layer to project raw inputs into a compressed 2048-dimensional representation $x_{m}^{\prime }\text{ to reduce computational complexity}$ , and then applies a learned attention mechanism in which these compressed features are weighted by a softmax-normalized score vector $a_{m}=softmax\left(W_{m}x_{m}+b_{m}\right)$ . An element-wise multiplication is applied to scale the feature vector by its corresponding attention weights, producing a weighted embedding $h_{m}=a_{m}⨀x_{m}^{\prime }$ . This mechanism functions as a sample-specific soft feature-gating layer that upweights compressed feature dimensions most informative for downstream reconstruction through the joint variational autoencoder (VAE). This allows the model to selectively emphasize informative features within each modality independently prior to any cross modal interaction. Following this independent gating step, these embeddings are concatenated to form a fused representation $h=\left[h_{GE}\;;\;h_{{DNA}_{meth}}\right]$ , which is passed to the joint VAE encoder. The gated representation is subsequently projected to a 512-dimensional modality-specific embedding. The two 512-dimensional embeddings are then concatenated to form a 1,024-dimensional fused representation, which is passed to the joint VAE encoder.

#### 2.2.2 Joint VAE Encoder

The joint encoder consists of six stacked fully connected layers with ReLU activations that process the fused representation h. The final layer of this shared network parameterizes a Gaussian latent distribution, $q_{\phi }\;\left(z\left.\;\right|x\right)=N\left(\mu \left(h\right),diag\left(\sigma^{2}\left(h\right)\right)\right)$ , where $\mu \left(h\right)$ and $\log\left(\sigma^{2}\left(h\right)\right)$ represent the learned mean and variance vectors used to sample the shared latent representation z.

#### 2.2.3 Modality-specific VAE decoders

To reconstruct each input modality independently, STRIVE routes the shared latent samples **z** to two separate, modality-specific decoders. Each individual decoder consists of six stacked fully connected layers, mirroring the depth of the joint VAE encoder network, mapping the shared latent representation **z** obtained from the Joint VAE encoder back to the original input dimensions for each modality independently:

$${\hat{x}}_{GE}={Decoder}_{GE}\left(z\right),{\hat{x}}_{{DNA}_{meth}}={Decoder}_{{DNA}_{meth}}\left(z\right)$$

where ${\hat{x}}_{GE}$ and ${\hat{x}}_{{DNA}_{meth}}$ represent the reconstructed feature vectors for gene expression and DNA methylation, respectively.

This parallel decoding architecture encourages the final learned latent representation to retain information relevant to both data modalities. This design follows established precedent in multimodal omics clustering. Lin et al. (2022) pair a single encoder over concatenated multi-omic inputs with a separate decoder for each modality in their scMDC framework, and Rockel et al. (2025) adopt an analogous structure in omicVAE, coupling one shared encoder to four modality-specific decoders so that modality-specific features are preserved while shared latent information is leveraged, identifying domain-specific reconstruction as critical to clustering tasks of this kind.

It is worth distinguishing two related but distinct properties of the shared latent representation z. The first is the preservation of modality-specific information: because each decoder must reconstruct its own modality from z alone, the encoder is penalised whenever z discards information required by either modality, so the two reconstruction terms are only jointly minimised when z retains signal from both gene expression and DNA methylation alongside the structure shared between them. The second is disentanglement, whether the latent dimensions are mutually independent, and whether modality-specific and shared signal are allocated to separate coordinates. STRIVE’s architecture achieves the former but not the latter: its objective contains no explicit independence or total-correlation penalty (β = 1, corresponding to the standard evidence lower bound), and the latent space is not partitioned into private and shared subspaces. The parallel decoders therefore encourage modality-specific signal to be retained within z without separating that signal into dedicated coordinates, reflecting the design goal of a single integrated coordinate system in which patient subtypes are defined by position within the latent space as a whole, rather than by a set of separable generative factors.

#### 2.2.4 Objective function

STRIVE was trained by minimizing a composite objective that balances reconstruction fidelity with latent space regularization, with the reconstruction and KL terms weighted equally:

$$L_{total}=L_{rec}+L_{KL}$$

$$L_{rec}=\frac{1}{N_{GE}}\sum_{i=1}^{N}{\left\|x_{GE}^{\left(i\right)}-{\hat{x}}_{GE}^{\left(i\right)}\right\|}^{2}+\frac{1}{N_{{DNA}_{meth}}}\sum_{i=1}^{N}{\left\|x_{{DNA}_{meth}}^{\left(i\right)}-{\hat{x}}_{{DNA}_{meth}}^{\left(i\right)}\right\|}^{2}$$

$$L_{KL}=D_{KL}\left(q_{\phi }\left(z\;|\;x\right)\;\|\;p\left(z\right)\;\right),\text{where }p\left(z\right)=N\left(0,I\right)$$

The reconstruction loss, $L_{rec}$ , is computed across both independent modalities as the mean squared error between the original input profiles $\left\{x_{GE},x_{{DNA}_{meth}}\right\}$ and their corresponding reconstructed outputs $\left\{{\hat{x}}_{GE},{\hat{x}}_{{DNA}_{meth}}\right\}$ generated by the decoders. This formulation forces the shared encoder and preceding attention blocks to retain features critical to both modalities.

Each term in $L_{rec}$ is normalised by the number of features in that modality so that the loss reflects the mean squared error per feature rather than the total summed error. This normalisation is necessary because the DNA methylation profiles used here are substantially higher-dimensional than the gene expression profiles: without it, an unweighted sum would let the methylation term dominate the gradient in proportion to its feature count rather than its informativeness, biasing the shared encoder and attention blocks toward methylation-derived structure at the expense of gene expression. The per-feature mean therefore balances the contribution of each modality to $L_{rec}$ without requiring an additional, manually tuned modality-weighting coefficient.

The KL divergence, $L_{KL}$ , regularizes the latent space toward the prior, acting as the central regularizer that binds the encoders and decoders together.

No additional regularisation term is applied directly to the attention weights $a_{m}$ beyond the softmax activation itself, which constrains each $a_{m}$ to be non-negative and to sum to 1 across the compressed feature dimension. Attention is instead learned implicitly: because $a_{m}$ lies on the computational path between the raw input $x_{m}$ and the reconstruction loss $L_{rec}$ , its values are shaped only by their effect on reconstruction fidelity and latent regularisation, rather than being constrained toward any pre-specified sparsity or distributional target.

Together $L_{rec}$ and $L_{KL}$ coordinate the interaction between the individual attention gates, the shared encoder, and the two separate decoders, encouraging STRIVE to learn compact latent embeddings that preserve multimodal signal while improving generalization. This design preserves feature-level interpretability through the attention weights while maintaining clustering performance comparable to non-attention and linear integration baselines (Supplementary Note S2).

STRIVE’s parameter count and computational cost scale approximately linearly with the number of modalities: each additional modality introduces its own attention block (a feature-compression layer and an attention-scoring layer) and its own decoder, so growth in model size is additive rather than combinatorial as further modalities are incorporated. Latent dimensionality is fixed independently of the number of modalities; the joint encoder’s input width, by contrast, scales with the number of modalities, since it operates on the concatenation of each modality’s attention-derived encoding, though its depth and overall design remain unchanged. Within a given modality, the dominant computational cost arises from the initial feature-compression layer, whose parameter count scales with that modality’s raw input dimension (input dimension × 2048 units); the downstream joint encoder and decoders instead operate on fixed-width representations (2048, 512, and 1024 dimensions in the two-modality configuration used here) regardless of the raw feature count. Increases in sample size accordingly affect training time, through additional forward and backward passes per epoch, rather than model size. Formal runtime and scalability benchmarking across a larger number of modalities was outside the scope of this study; the analysis above characterises expected computational behaviour based on the model architecture rather than empirical measurement.

### 2.3 Multimodal signature identification

We applied K-means clustering to the final learned latent representation, **z,** obtained from STRIVE to identify molecular subtypes within each cohort. The optimal number of clusters was selected using standard cluster validity diagnostics (e.g., scree and silhouette analyses). Following this identification of latent clusters, we employed three analytical approaches: (i) encoder-weight based feature contributions, (ii) gene signature identification, and (iii) permutation feature importance (PFI). These approaches are complementary and address distinct questions. The encoder weight analysis (Section 2.3.1) identifies which input features most shape the STRIVE latent representation on which the clustering is based; it is unsupervised with respect to both cluster label and survival, yields a single cohort-wide feature ranking, and reduces the full feature space to a compact, model-informed panel for downstream predictive analysis. The gene signature identification (Section 2.3.2) instead characterizes the molecular identity of each subtype by isolating features that are specifically elevated in one cluster relative to all others; it uses cluster membership but not survival, and is applied to the full feature set independently of the encoder-weight ranking. The PFI analysis (Section 2.3.3) quantifies the prognostic relevance of features within each subtype by measuring how much each feature contributes to predicting survival; it uses the survival outcome and is applied to the encoder-weight derived panel. In brief, the three analyses respectively identify the features that shape the latent representation, the features that define each subtype, and the features that predict outcome within each subtype.

#### 2.3.1 Encoder weight based feature contributions

Encoder-weight based feature contributions analysis establishes which raw input features most influence the low-dimensional latent representation that STRIVE learns and on which the clustering is performed.

To attribute variation in the STRIVE latent space to individual input features, we extracted the weight matrix of each encoder layer and combined them by successive dot products into a single composite operator, which was then applied to each modality. The per-layer weight matrices of the shared joint encoder were multiplied together to give one operator mapping the concatenated 1,024-dimensional embedding onto the three-dimensional latent space; because this embedding concatenates the two 512-dimensional modality embeddings, the composite operator was split row-wise into a gene-expression block and a DNA-methylation block. Each modality also has its own encoder, whose feature-compression and projection matrices were multiplied together into a composite mapping the raw features of that modality onto its 512-dimensional embedding. Multiplying this modality composite by the corresponding block of the joint operator then chains the two stages into a single operator that maps the raw gene-expression or DNA-methylation features directly onto the three latent dimensions. To score features, each sample’s latent representation was back-projected through the transpose of this operator, producing a sample-by-feature contribution matrix; each feature’s importance was defined as its mean absolute contribution across all samples, and features were ranked by this score to prioritize the most influential genes and methylation sites for downstream analysis. The procedure is a first-order linear approximation, since multiplying the layer weight matrices directly omits the intervening nonlinear activations and input-dependent attention weighting. This ranking is unsupervised, reflecting only the geometry of the learned representation and using neither cluster labels nor survival status. It serves two roles: it highlights the genes and methylation features that most govern the latent space, and it defines the compact, outcome-independent candidate panel (the top-ranked features per modality) that is carried forward to the predictive analysis in Section 2.3.3.

#### 2.3.2 Gene signature identification within each cluster

While the encoder-weight analysis ranks features by their influence on the latent representation as a whole and is blind to cluster membership, the gene signature analysis asks which features distingush one subtype from the others, thereby characterizing the molecular identity of each cluster rather than the drivers of the representation. It was applied to the full standardized feature set rather than the encoder-weight panel, so that subtype signatures are not restricted to the features that dominate the latent space.

Gene-expression and DNA-methylation features were standardized prior to analysis, so that differences between cluster means correspond to standardized mean differences. To identify signature features driving cluster differentiation, we then performed a standardized mean difference (SMD) analysis, testing each STRIVE cluster against all remaining samples in a one-vs-rest design (rather than all pairwise cluster pairs). Significance was assessed with a linear model fit in limma using empirical Bayes moderation of the variance, and features were retained at Benjamini–Hochberg-adjusted q < 0.05. To ensure markers were specifically upregulated in the target cluster, we further required that the target cluster’s standardized mean exceed both the pooled mean of all other clusters and the mean of every individual other cluster. After BH correction and application of this directional-consistency criterion, 823, 561, 345, 121, 230, and 510 features qualified for clusters 1–6, respectively (2,590 cluster-specific significant features overall). Features satisfying these criteria were ranked by adjusted significance, and the top 10 features per cluster were retained as the SMD-based signatures for visualization and biological interpretation.

#### 2.3.3 Permutation feature importance for predictive relevance

The two preceding analyses are agnostic to the clinical outcome: encoder-weight contributions reflect the structure of the latent representation and SMD signatures reflect molecular differences between sub-types, but neither indicates whether a feature carries prognostic information. This analysis closes that gap by quantifying within each subtype, how much each feature contributes to predicting survival. This exploratory analysis therefore examines, within each STRIVE-derived cluster, which molecular features contribute most to predicting survival status. To identify features most predictive of survival within each STRIVE-derived cluster, we implemented a permutation feature importance (PFI) framework using four classification models: logistic regression, random forest, standard gradient boosting (gradient boosting machine, GBM), and extreme gradient boosting (XGBoost). The candidate feature panel entering this framework comprised the top-ranked gene expression and DNA methylation features derived from STRIVE encoder weight based feature contributions. This upstream ranking was unsupervised with respect to survival status and therefore did not incorporate the survival outcome used in the downstream prediction task. For each cluster, samples were first partitioned into training and test sets using a stratified 70/30 hold-out split (stratified on survival status). To avoid data leakage, all downstream preprocessing, class balancing, feature-importance computation, and PFI-based feature selection were performed exclusively on the training partition; the test set was reserved solely for final performance estimation.

Within the training data only, features were standardized (z-scored) and the class distribution was balanced using the Synthetic Minority Over-sampling Technique (SMOTE). SMOTE mitigates class imbalance by generating synthetic minority-class examples through interpolation between existing minority-class samples and their nearest neighbours, rather than by simple duplication of existing cases. Balancing was necessary because survival events were unequally distributed across the binary survival outcome within clusters, which would otherwise bias the classifiers toward the majority class; test-set scaling parameters were derived from the training data, and SMOTE was never applied to the test set.

An initial classifier was trained on the balanced training data, and PFI was computed on the training partition by quantifying the reduction in model performance (ROC-AUC) when each feature was independently permuted (five permutation repeats per feature). Features producing larger performance reductions were assigned higher PFI scores. The top 50 features per cluster were selected on the basis of these training-derived PFI scores, and a second classifier was retrained on this subset, again using training data only. Final predictive utility was evaluated on the held-out test set using ROC-AUC, with 95% confidence intervals estimated by bootstrap resampling (1,000 iterations). Because the train/test split preceded all scaling, resampling, feature-importance estimation, and feature selection, the reported test-set AUCs are not subject to leakage from these steps. These within-cluster analyses were considered exploratory and were not used to validate the STRIVE-derived clusters themselves.

## 3 Results

Given the BRCA cohort size and primary study focus, we present BRCA results in the main manuscript, with replication analyses in UCEC and CESC reported in the Supplementary Material.

### 3.1 Subtype discovery and multimodal signature characterization using STRIVE

To investigate molecular heterogeneity, we first analyzed 866 breast cancer (BRCA) samples with matched gene expression, DNA methylation, survival, and phenotype data. Cohort characteristics are summarized in Table 1.

**TABLE 1:** Summary statistics for breast cancer cohort from TCGA-BRCA.

Characteristics	Full sample (N = 866)
Survival proportion	0.844
Survival time (months)	
Mean (SD)	41.7 (37.2)
Median (Q1, Q3)	29.8 (16.6, 55.6)
Sex, n (%)	
Female	857 (99)
Male	9 (1)
Age (years)	
Mean (SD)	57.7 (13.0)
Median (Q1,Q3)	58 (48, 67)
Race, n (%)	
White	649 (74.9)
Black | African American	162 (18.7)
Asian	38 (4.4)
Other	1 (0.1)
Missing, n	16
AJCC pathologic stage, n (%)	
I	137 (15.8)
II	489 (56.5)
III	216 (24.9)
IV	12 (1.4)
Unknown, n	12
Primary tumor size (T), n (%)	
Small (≤2 cm)	217 (25.1)
Medium (>2 cm but ≤ 5 cm)	499 (57.6)
Large (>5 cm)	119 (13.7)
Locally advanced/invasive	28 (3.2)
Unknown, n	3
Lymph node involvement (N), n (%)	
None (N0)	381 (44.0)
Low (N1)	309 (35.7)
Moderate (N2)	104 (12.0)
Extensive (N3)	60 (6.9)
Unknown, n	12
Metastasis status (M), n (%)	
No distant metastasis (M0)	692 (79.9)
Distant metastasis present (M1)	14 (1.6)
Unknown, n	160
Tumor histologic type, n (%)	
Ductal and lobular neoplasms	825 (95.3)
Cystic, mucinous, serous neoplasms	15 (1.7)
Complex epithelial neoplasms	15 (1.7)
Epithelial neoplasms	5 (0.6)
Fibroepithelial neoplasms	1 (0.1)
Adenomas and adenocarcinomas	2 (0.2)
Squamous cell neoplasms	2 (0.2)
Basal cell neoplasms	1 (0.1)
Prior malignancy, n (%)	
No	814 (94.0)
Yes	51 (5.9)
Unknown, n	1

Following preprocessing, the analytic feature space comprised 344,743 molecular variables spanning two domains: 44,341 gene expression and 300,402 DNA methylation features. We then applied STRIVE with K-means clustering on the learned latent representation, identifying six molecular clusters (subtypes) in BRCA (Figure 2). The choice of k = 6 was supported by scree and silhouette analyses, which showed a marked change in explained variance and supported jointly by the scree elbow and silhouette diagnostics (Supplementary Figure S1; Supplementary Note S3).

**FIGURE 2:**
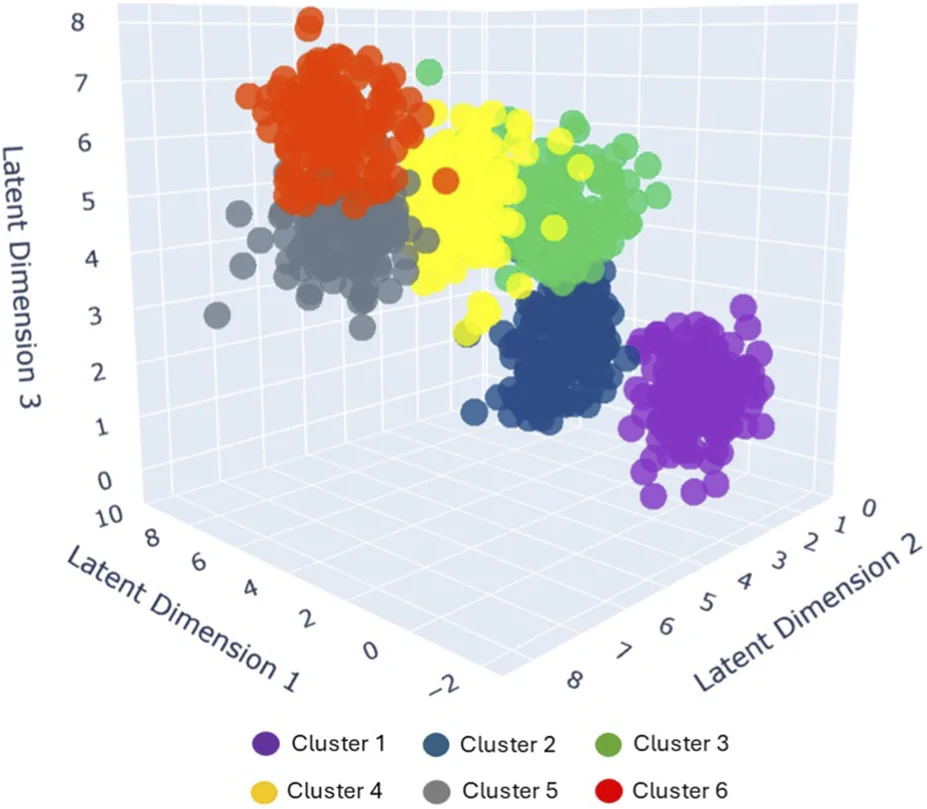
K-Means Clustering of Integrated Molecular Features using STRIVE-Guided Latent Embeddings. K-means clustering was applied to the learned latent embedding space, identifying six patient clusters represented in a 3D projection. Each point corresponds to an individual patient, with colors indicating cluster assignment: Three-dimensional illustration of 6 patient clusters identified using the STRIVE framework. Clusters include: Cluster 1 (purple, n = 154), Cluster 2 (blue, n = 141), Cluster 3 (green, n = 137), Cluster 4 (yellow, n = 141), Cluster 5 (grey, n = 138), and Cluster 6 (red, n = 155). The clustering pattern highlights the molecular heterogeneity captured by the STRIVE-derived latent representations.

Survival outcomes and clinicopathological characteristics by cluster are summarized in Table 2. Cluster sizes were broadly comparable, with survival proportions ranging from 79.7% to 89.4% and median survival times ranging from 25.0 to 33.7 months. Statistical testing indicated no significant differences in survival proportion (Chi-squared test) or survival time (Kruskal–Wallis test) across the six clusters. Accordingly, the identified clusters were not interpreted as clinically or prognostically validated subtypes on the basis of overall survival. Among pathological characteristics, only AJCC stage (*p* = 0.032) and tumor size (T; *p* = 0.023) differed across clusters; these concordant effects reflect a single tumor-burden axis, with clusters 4–5 enriched for larger, later-stage tumors and clusters 1–3 for small, early-stage disease. These findings were interpreted cautiously given the multiple clinicopathological comparisons and sparse categories and did not track with survival. We therefore interpret these as suggestive of a partial link to tumor burden rather than a robust clinicopathological determinant of the clusters.

**TABLE 2:** Survival outcomes, clinical and pathological characteristics by cluster for TCGA-BRCA.

Characteristics	Cluster 1	Cluster 2	Cluster 3	Cluster 4	Cluster 5	Cluster 6	P-value
Sample size	154	141	137	141	138	155	
Survival proportion	0.864	0.837	0.861	0.894	0.797	0.813	0.228
Survival time (months)							
Mean (SD)	42.7 (40.8)	39.3 (36.9)	43.5 (38.1)	40.1 (39.0)	41.7 (33.5)	43.0 (34.9)	0.641
Median (Q1, Q3)	26.7 (16.3, 59.3)	27.8 (17.4, 51.1)	33.7 (17.2, 53.8)	25.0 (15.3, 51.5)	30.9 (16.3, 58.6)	33.2 (18.5, 60.8)	
AJCC pathologic stage, n (%)^a^							0.032
I	24 (15.8)	34 (24.3)	27 (20.1)	19 (13.6)	11 (8.1)	22 (14.4)	
II	91 (59.9)	64 (45.7)	71 (53.0)	83 (59.3)	93 (68.9)	87 (56.9)	
III	35 (23.0)	41 (29.3)	35 (26.1)	37 (26.4)	28 (20.7)	40 (26.1)	
IV	2 (1.3)	1 (0.7)	1 (0.7)	1 (0.7)	3 (2.2)	4 (2.6)	
Primary tumor size (T), n (%)^a^							0.023
Small (≤2 cm)	41 (26.8)	48 (34.0)	44 (32.4)	26 (18.4)	26 (19.0)	32 (20.6)	
Medium (>2 cm but ≤5 cm)	85 (55.6)	71 (50.4)	67 (49.3)	88 (62.4)	95 (69.3)	93 (60.0)	
Large (>5 cm)	22 (14.4)	16 (11.3)	22 (16.2)	24 (17.0)	11 (8.0)	24 (15.5)	
Locally advanced/invasive	5 (3.3)	6 (4.3)	3 (2.2)	3 (2.1)	5 (3.6)	6 (3.9)	
Lymph node involvement (N), n (%)							0.947
None (N0)	67 (44.1)	56 (40.6)	58 (43.0)	65 (46.4)	63 (46.7)	72 (46.8)	
Low (N1)	57 (37.5)	50 (36.2)	51 (37.8)	47 (33.6)	52 (38.5)	52 (33.8)	
Moderate (N2)	19 (12.5)	18 (13.0)	16 (11.9)	18 (12.9)	11 (8.1)	22 (14.3)	
Extensive (N3)	9 (5.9)	14 (10.1)	10 (7.4)	10 (7.1)	9 (6.7)	8 (5.2)	
Metastasis status (M), n (%)^a^							0.482
No distant metastasis (M0)	126 (98.4)	115 (99.1)	108 (99.1)	114 (98.3)	110 (97.3)	119 (96.0)	
Distant metastasis present (M1)	2 (1.6)	1 (0.9)	1 (0.9)	2 (1.7)	3 (2.7)	5 (4.0)	
Tumor histologic type, n (%)^a^							0.599
Adenomas and adenocarcinomas	1 (0.6)	0 (0.0)	1 (0.7)	0 (0.0)	0 (0.0)	0 (0.0)	
Basal cell neoplasms	0 (0.0)	0 (0.0)	0 (0.0)	0 (0.0)	1 (0.7)	0 (0.0)	
Complex epithelial neoplasms	0 (0.0)	2 (1.4)	1 (0.7)	5 (3.5)	3 (2.2)	4 (2.6)	
Cystic, mucinous and serous neoplasms	3 (1.9)	1 (0.7)	4 (2.9)	3 (2.1)	3 (2.2)	1 (0.6)	
Ductal and lobular neoplasms	149 (96.8)	137 (97.2)	129 (94.2)	131 (92.9)	130 (94.2)	149 (96.1)	
Epithelial neoplasms, NOS	0 (0.0)	1 (0.7)	2 (1.5)	1 (0.7)	0 (0.0)	1 (0.6)	
Fibroepithelial neoplasms	1 (0.6)	0 (0.0)	0 (0.0)	0 (0.0)	0 (0.0)	0 (0.0)	
Squamous cell neoplasms	0 (0.0)	0 (0.0)	0 (0.0)	1 (0.7)	1 (0.7)	0 (0.0)	
Prior malignancy, n (%)							0.863
No	145 (94.2)	134 (95.7)	128 (93.4)	133 (94.3)	131 (94.9)	143 (92.3)	
Yes	9 (5.8)	6 (4.3)	9 (6.6)	8 (5.7)	7 (5.1)	12 (7.7)	
Tissue source site, n (%)^a,b^							0.363

Differences across clusters were assessed using Pearson’s chi-square test for categorical variables and the Kruskal–Wallis test for survival time.

^a^ For categorical variables in which more than 20% of cells had an expected count below 5, the chi-square approximation is unreliable and a Fisher–Freeman–Halton exact test was used (indicated by ^a^).

^b^ Tissue source site was evaluated as a proxy for source-related technical variation; low-frequency tissue source sites were pooled into an “Other” category prior to testing.

All p-values are two-sided and reported without adjustment for multiple comparisons.

To characterize cluster-defining molecular signals, we derived distinct multimodal signatures for clusters 1–6 using standardized mean difference (SMD) analyses across gene expression and DNA methylation features (q < 0.05; Figure 3). Notably, all 10 of the top-ranked cluster-defining features for clusters 3 and 5 were DNA-methylation features, whereas the top-ranked signatures for the remaining clusters spanned both molecular domains. The most prominent differentiating features included AZU1-associated CpGs (cg02147126, cg15610437, cg16643542) for cluster 1; GPR135 and L3HYPDH (cg04329105) for cluster 2; HOXA-AS2 and HOXA3 (cg22962123) for cluster 3; F2 (cg18868540) for cluster 4; and KIFC2 (cg22614142) for cluster 6. In contrast, cluster 5 exhibited comparatively homogeneous feature values, without a single dominant marker.

**FIGURE 3:**
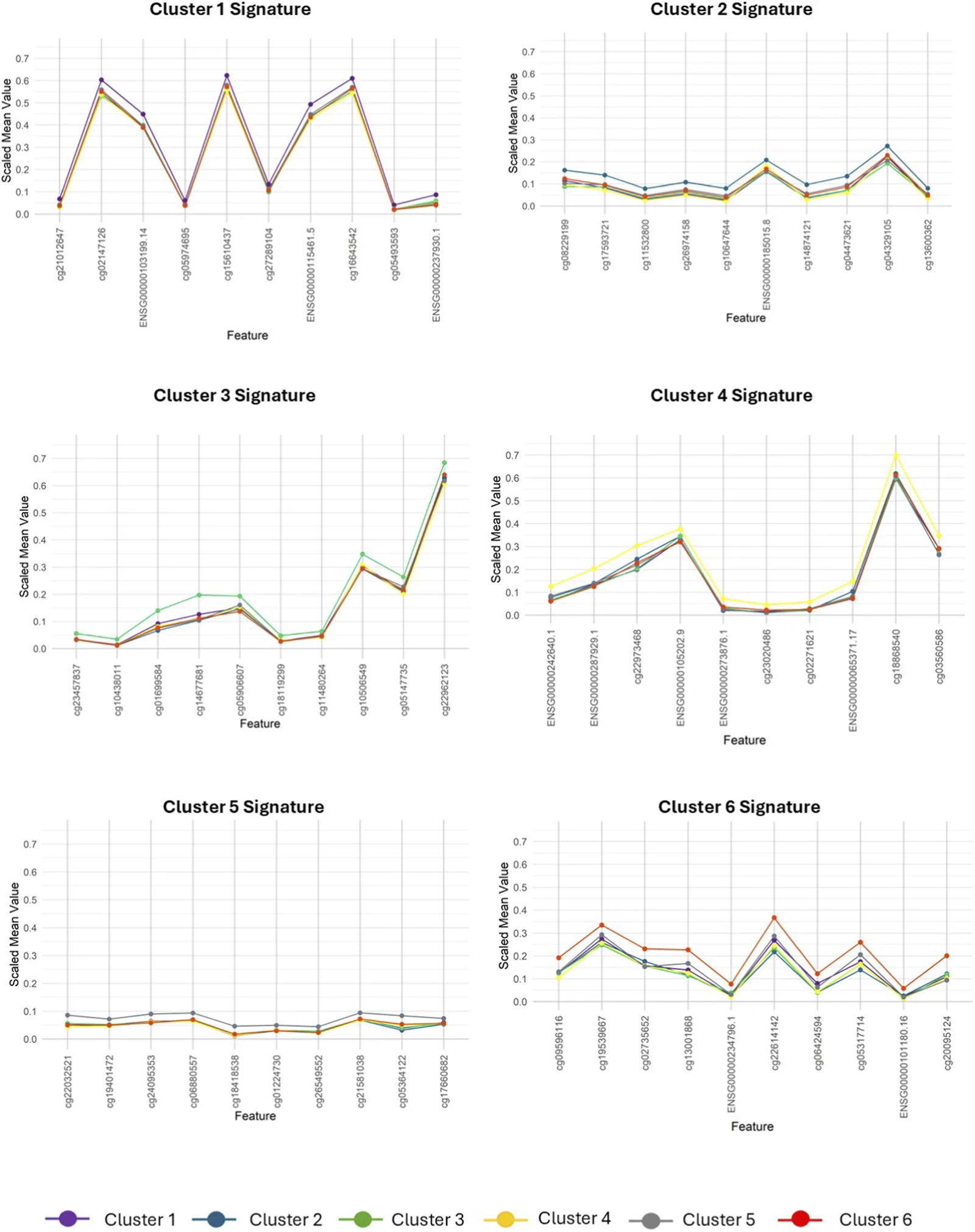
Integrated analysis of patient clustering and gene expression and DNA feature signatures. Molecular signature profiles for each of the six STRIVE-derived clusters, identified using standardized mean difference (SMD) analysis (q < 0.05) across gene expression and DNA methylation domains. Features were retained as cluster-specific markers only if consistently elevated in the target cluster relative to all others. DNA methylation probes are identified by CpG identifiers and gene expression features by gene symbols. Notably, clusters 3 and 5 were defined exclusively by DNA methylation features, while remaining clusters contained signatures spanning both modalities. The x-axis shows standardized mean differences, with larger values indicating stronger cluster differentiation.

### 3.2 Survival prediction within clusters using permutation feature importance

To identify features most predictive of survival within each STRIVE-derived cluster, we implemented a permutation feature importance (PFI) framework across multiple classifiers (logistic regression, random forest, and gradient boosting variants). Model performance was evaluated on held-out test data using ROC-AUC (Table 3).

**TABLE 3:** Mean area under the receiver operating characteristic curve (AUC) for each classifier evaluated within each STRIVE-derived cluster for TCGA-BRCA.

Models	Cluster 1	Cluster 2	Cluster 3	Cluster 4	Cluster 5	Cluster 6
XGBoost	0.520	0.528	0.546	**0.711**	0.502	0.576
Gradient boosting	0.608	0.563	0.623	**0.761**	0.407	0.482
Random forest	**0.663**	0.556	0.532	**0.663**	0.586	0.635
Logistic regression	**0.638**	0.429	0.579	0.537	0.406	0.484

Performance was evaluated on a held-out test set following a single stratified 70/30 train/test split. Bold values indicate the highest performing classifier within each cluster.

Exploratory ROC-AUC performance varied across clusters and classifiers, with wide bootstrap 95% confidence intervals indicating substantial uncertainty (Table 3). Although some ensemble models produced higher point estimates in selected clusters, these comparisons are presented as sensitivity analyses rather than evidence of definitive predictive performance.

### 3.3 Validation analysis in additional cancer cohorts

To assess whether STRIVE’s stratification and signature findings generalize to additional cohorts with smaller sample sizes, we applied the same pipeline to 454 endometrial cancer (UCEC) and 296 cervical cancer (CESC) samples. Results are presented in Supplementary Tables S1–S6 and Supplementary Figures S2–S7.

Supplementary Tables S1, S2 summarize the UCEC and CESC cohorts. Compared with BRCA, UCEC and CESC exhibited lower survival proportions (82.8% and 75.0%, respectively) and shorter mean survival times (33.3 ± 28.1 months and 35.1 ± 37.5 months, respectively). UCEC patients were older on average (64.0 ± 11.2 years), whereas CESC patients were younger (48.1 ± 13.8 years), relative to BRCA. Racial composition across cohorts followed a similar pattern, with most patients identifying as White, followed by Black or African American and Asian individuals. Prior malignancy was more common in UCEC (8.8%) and less frequent in CESC (2.7%) relative to BRCA (5.9%).

For both UCEC and CESC, four clusters were selected as optimal (Supplementary Figures S2–S5). Cluster sizes were generally balanced, with survival proportions ranging from 77.9% to 87.4% in UCEC and 68.3%–81.5% in CESC. As in BRCA, survival outcomes did not differ significantly across clusters in either cohort (Supplementary Tables S3, S4).

Signature composition differed by cohort. In CESC, the signature for cluster 1 consisted exclusively of DNA methylation features, whereas the remaining clusters across UCEC and CESC generally reflected a mixture of gene expression and DNA methylation features (Supplementary Figures S6, S7). In UCEC, the most prominent defining features included LDLR (cg24113439) for cluster 1, TNRC18 (cg04939944) for cluster 2, THBS3 (ENSG00000169231.13) for cluster 3, and cg1775212 and PSMB3 (cg04409945) with similar mean expression patterns in cluster 4. In CESC, cluster 1 was dominated by cg09116625; cluster 2 by NIBAN2 (ENSG00000136830.12); cluster 3 showed comparatively homogeneous feature values; and cluster 4 showed higher values for LDHC (ENSG00000166796.12), DHRS7, and PCNX4 (cg15876689), as well as AL050403.2 and JAG1 (cg20319775).

In survival prediction, XGBoost again showed the most consistent performance in UCEC, with AUC > 0.5 across all four clusters (Supplementary Table S5). In contrast, predictive performance in CESC was variable, with multiple clusters yielding AUC < 0.5 and no single classifier consistently outperforming others (Supplementary Table S6), suggesting weaker or more heterogeneous survival signal in this cohort.

## 4 Discussion

Across breast, endometrial, and cervical cancers, DNA-methylation features were prominently represented among several cluster-defining signatures. STRIVE integrates these complementary transcriptomic and epigenomic signals using modality-specific feature processing before fusion, without explicitly weighting one modality against the other. This design provides a flexible framework for integrating complementary molecular information and has an immediate implication for how the field approaches multi-omic subtype discovery. Gene expression has historically dominated cancer stratification pipelines, yet the results presented here suggest that this prioritization may miss some epigenomically defined patient groups whose regulatory programs are biologically distinct but transcriptomically diffuse. STRIVE was designed to address this limitation by learning modality-aware embeddings before fusion and preserving complementary transcriptomic and epigenomic information within a shared latent representation. The discussion that follows interprets these findings in the context of what they reveal about the structure of molecular heterogeneity in women’s cancers and what they imply for the design of future multi-omic stratification frameworks.

In TCGA-BRCA, STRIVE identified six subtypes with distinct multimodal signatures. Across cohorts, DNA-methylation features were prominently represented among several of the strongest cluster-defining signatures. However, because substantially more DNA-methylation than gene-expression features were available in the candidate feature space, this pattern may partly reflect modality dimensionality and should not be interpreted as evidence that methylation globally dominates the STRIVE latent representation. Rather, the findings demonstrate that the integrated representation captures complementary transcriptional and epigenomic structure. The presence of CpG-level features among several cluster-specific signatures suggests that DNA methylation contributes complementary information to patient stratification that may not be fully reflected by gene expression alone. These representative signatures also link the STRIVE-derived clusters to specific annotated genes and regulatory regions, including HOXA-AS2/HOXA3, F2, and KIFC2-associated loci. We interpret these features as candidate molecular markers of cluster-specific biology rather than established mechanistic drivers, and their functional relevance will require further experimental validation.

Across cohorts, overall survival did not differ significantly across the STRIVE-derived clusters. Within-cluster survival-status prediction showed variable performance across clusters and classifiers, with relatively wide uncertainty in several estimates. Given the reduced sample and event counts within individual clusters, these analyses should be interpreted as exploratory and hypothesis-generating rather than as evidence of prognostic validity or subtype-specific predictive utility. Their primary value in the present study is to identify candidate outcome-associated molecular features that may warrant evaluation in larger independent cohorts.

Replication analyses in UCEC and CESC demonstrated that STRIVE can recover structured latent subgroups and multimodal signatures in smaller cohorts, with DNA-methylation features again prominently represented in specific cluster signatures. The reduced stability of survival prediction in CESC highlights an important consideration for multimodal outcome modeling in practice. More broadly, when sample sizes are limited and outcomes are heterogeneous, survival signal may be weak relative to molecular and technical variability, and performance may depend strongly on endpoint definition, follow-up, and cohort composition. These observations motivate future applications that incorporate richer clinical endpoints beyond overall survival, including progression-free survival, recurrence, treatment response, or therapy-specific outcomes where molecular stratification may translate more directly into clinically actionable differences.

A few limitations and natural extensions should be noted. Our primary outcome assessment focused on overall survival, which is influenced by treatment heterogeneity and follow-up variability and may be relatively insensitive to subtype-specific clinical differences. In addition, the current analysis integrates two omics layers, whereas incorporating additional modalities such as copy number alterations, somatic mutations, proteomics, or imaging could capture further sources of heterogeneity. Cluster characterization was additionally limited by the availability and completeness of established molecular and clinicopathological annotations, particularly in the smaller UCEC and CESC cohorts. Finally, replication in smaller cohorts suggests that predictive performance can be cohort-dependent, particularly when sample size and event counts are limited. Future work will extend STRIVE to richer phenotypes including recurrence, progression-free survival, and therapy response, incorporate treatment and microenvironment covariates to improve clinical interpretability, and evaluate external validation cohorts to strengthen generalizability. More tightly coupling stratification with outcome modeling, for example, via subtype-specific survival models or mixture-of-experts approaches, may further improve identification of clinically actionable subgroups.

In summary, STRIVE provides an interpretable, attention-weighted framework for jointly modeling transcriptomic and epigenomic heterogeneity in women’s cancers. Across all three cohorts, DNA methylation features were prominently represented among cluster-defining signatures, even within a framework that integrates both modalities simultaneously and without prior weighting toward either. This pattern carries a practical implication that extends beyond STRIVE itself: subtype-discovery pipelines that rely on gene expression alone may miss some patient groups characterized by distinct epigenomic regulatory programs that are not fully reflected at the transcriptomic level. The recurrence of methylation-rich signatures across breast, endometrial, and cervical cancers suggests that epigenomic information contributes complementary structure to multi-omic cancer heterogeneity, although the larger methylation candidate feature space should be considered when interpreting this pattern. STRIVE establishes a practical foundation for multimodal molecular stratification and future evaluation of subtype-specific prognostic associations and points toward a broader consideration: in multi-omic cancer stratification, epigenomic signals should be considered alongside transcriptomic signals, and frameworks that integrate both modalities are better positioned to reveal complementary molecular disease structure.

## Data Availability

The datasets analyzed for this study are publicly available and can be found in The Cancer Genome Atlas (TCGA) repositories for breast, endometrial, and cervical cancer (Goldman et al., 2020). Analysis code is available on GitHub at https://github.com/zunairamehmood/strive.
