# Supplementary material for: STRIVE: interpretable multimodal subtype discovery in Women’s cancers using an attention-weighted joint variational autoencoder
Source: Front Bioinform. 2026 Sep 29;6:1901464. doi: 10.3389/fbinf.2026.1901464 (PMC13658565; doi:10.3389/fbinf.2026.1901464)
Supplement: Supplementary file 1 [file DataSheet1.pdf]

STRIVE: Interpretable Multimodal Subtype Discovery in Women's Cancers Using an Attention-Weighted Joint Variational Autoencoder

Zunaira Mehmood[1], Divya Sharma[2,3*]

[1]Department of Biostatistics, Dalla Lana School of Public Health, University of Toronto, Toronto, ON, Canada
[2]Department of Mathematics and Statistics at York University, North York, ON, Canada
[3]Department of Biostatistics, University Health Network, Toronto, ON, Canada

Supplementary Notes on Methods:

Note S1. Single Modality Concordance
Note S2. Model Benchmarking
Note S3. Selection of Optimal Clusters

Supplementary Tables:

Table S1. Summary statistics for endometrioid cancer cohort (TCGA-UCEC).
Table S2. Summary statistics for cervical cancer cohort (TCGA-CESC).
Table S3. Survival outcomes by cluster for TCGA-UCEC.
Table S4. Survival outcomes by cluster for TCGA-CESC.
Table S5. Mean Area Under the Receiver Operating Characteristic Curve (AUC) for each classifier evaluated within each STRIVE-derived cluster for TCGA-UCEC.
Table S6. Mean Area Under the Receiver Operating Characteristic Curve (AUC) for each classifier evaluated within each STRIVE-derived cluster for TCGA-CESC.
Table S7: Comparison of latent-space clustering quality and stability across multimodal integration and single-modality models on TCGA-BRCA at k = 6.

Supplementary Figures:

Figure S1. Internal cluster validation metrics for establishing the optimal number of clusters for TCGA-BRCA.
Figure S2. Internal cluster validation metrics for establishing the optimal number of clusters for TCGA-UCEC.
Figure S3. Internal cluster validation metrics for establishing the optimal number of clusters for TCGA-CESC.
Figure S4. K-Means Clustering of Integrated Molecular Features using STRIVE-Guided Latent Embeddings for TCGA-UCEC.
Figure S5. K-Means Clustering of Integrated Molecular Features using STRIVE-Guided Latent Embeddings for TCGA-CESC.
Figure S6. Integrated analysis of patient clustering and gene expression and DNA feature signatures for TCGA-UCEC.
Figure S7. Integrated analysis of patient clustering and gene expression and DNA feature signatures for TCGA-CESC.

Supplementary Notes on Methods

Note S1. Single-Modality Concordance

To assess the added value of multi-omics integration, we tested whether gene expression and DNA methylation independently support similar clustering structure in the BRCA cohort. Highly concordant single-modality partitions would suggest that either omics layer alone is largely sufficient to recover patient subtypes, weakening the rationale for joint integration; discordant structure, conversely, would indicate that the two modalities carry complementary information, motivating an integrated approach such as STRIVE.

We clustered gene expression and DNA methylation data independently, passing each modality separately through the same attention-based VAE architecture used in STRIVE, and applied k-means (k = 6) to each resulting single-modality latent representation. We then computed the Adjusted Rand Index (ARI) between the two resulting sets of cluster assignments to quantify their concordance.

The gene expression-only and DNA methylation-only partitions showed negligible concordance, with a cross-modal ARI of 0.000077 indistinguishable from the value expected under random cluster assignment. This near-zero ARI indicates that the two modalities, when clustered independently, recover largely discordant patient groupings rather than replicating a shared underlying subtype structure. This finding supports the rationale for joint integration: neither modality alone is sufficient to recover the structure identified when both are integrated, and an approach such as STRIVE, which learns a shared latent representation from both data types jointly, is needed to capture the complementary signal each contributes.

Note S2. Model Benchmarking

To assess the quality and stability of STRIVE-derived latent representations, we benchmarked STRIVE against five comparison models: an otherwise matched joint VAE with the modality-specific attention blocks removed (VAE, no attention), simple early concatenation, Multi-Omics Factor Analysis (MOFA), a gene-expression-only attention-based VAE (GE-only), and a DNA-methylation-only attention-based VAE (DNAm-only). The multimodal comparisons were used to assess the contribution of nonlinear representation learning and the feature-gating mechanism, whereas the single-modality models were included to evaluate the added value of jointly modeling gene-expression and DNA-methylation information.

All six models were applied to the TCGA-BRCA cohort, and each resulting representation was clustered using k-means at a common resolution of k=6 to ensure comparability. Cluster quality and stability were evaluated using six complementary criteria: intrinsic geometric quality (silhouette coefficient, Davies–Bouldin index, and Calinski–Harabasz index), partition stability under resampling (bootstrap adjusted Rand index [ARI] and consensus score [1−PAC]), and minimum cluster size as a diagnostic for potentially degenerate partitions. Point estimates and 95% confidence intervals were obtained by resampling 80% of patients without replacement over 1,000 iterations and re-clustering each subsample; consensus scores were obtained using an

independent consensus-clustering procedure comprising 20 repeats of 100 subsamples each. Results are summarized in Table S7.

At k=6, STRIVE and the otherwise matched VAE without attention showed nearly identical intrinsic clustering quality, with silhouette coefficients of 0.238 (95% CI [0.229, 0.245]) for both models and Davies–Bouldin indices of 1.114 and 1.115, respectively. Their stability estimates were also comparable, with bootstrap ARI values of 0.835 and 0.819 and consensus scores of 0.859 and 0.864, respectively. Simple concatenation produced broadly similar but slightly lower stability estimates, whereas MOFA showed poorer intrinsic clustering performance and substantially lower consensus stability. MOFA also produced a highly imbalanced partition, with a mean minimum cluster size of approximately 7 patients compared with approximately 100 patients for STRIVE, the no-attention VAE, and simple concatenation.

The single-modality models produced weaker cluster-quality and stability metrics overall than the multimodal STRIVE representation. The GE-only and DNAm-only models yielded silhouette coefficients of 0.205 and 0.219, respectively, compared with 0.238 for STRIVE, together with lower Calinski–Harabasz indices and consensus scores. These findings support the value of integrating complementary gene-expression and DNA-methylation information rather than relying on either modality alone. Importantly, however, STRIVE did not show a substantial clustering advantage over the otherwise matched VAE without attention. We therefore interpret the feature-gating mechanism primarily as providing **sample-specific feature reweighting before multimodal fusion while preserving clustering quality and stability comparable to the no-attention VAE**, rather than as independently improving clustering performance.

Note S3. Selection of Optimal Clusters

The number of clusters was selected independently for each cohort by joint assessment of scree and silhouette analyses (Supplementary Figure S1), applying a consistent criterion across datasets: the scree elbow was taken as the primary indicator of parsimonious latent structure, and silhouette scores were used as a corroborating diagnostic rather than maximized in isolation.

TCGA-BRCA. The scree plot showed diminishing reductions in within-cluster variance beyond six clusters, consistent with an elbow at k = 6. Although silhouette scores were marginally higher at k = 23, 26, and 27 (ss = 0.238–0.241) than at k = 6 (ss = 0.2375), these local maxima were not accompanied by a corresponding inflection in the scree plot and yielded far less parsimonious solutions. A six-cluster solution was therefore selected as the partition best supported by both diagnostics (Supplementary Figure S1).

TCGA-UCEC. The scree plot indicated an elbow at k = 4, with a silhouette score of 0.244 at that resolution. Higher silhouette values occurred at substantially larger cluster numbers (ss = 0.249 at k = 15–16, 0.257 at k = 22, and 0.253 at k = 28), but as in BRCA these maxima were not reflected in the scree plot and corresponded to markedly less parsimonious partitions. A four-cluster solution was therefore selected (Supplementary Figure S2).

TCGA-CESC. Both diagnostics were concordant, with a scree elbow at k = 4 and the silhouette score also maximized at that resolution (ss = 0.259). A four-cluster solution was therefore selected as the partition jointly supported by both criteria (Supplementary Figure S3).

Supplementary Tables

Table S1: Summary statistics for endometrioid cancer cohort (TCGA-UCEC)

	**Full sample (N = 454)**
**Survival proportion**	0.828
**Survival time (months)**	
Mean (SD)	33.3 (28.1)
Median (Q1, Q3)	26.3 (15.4, 43.7)
**Age (years)**	
Mean (SD)	64.0 (11.2)
Median (Q1,Q3)	64 (57, 71)
**Race**, n(%)	
White	307 (67.6)
Black | African American	101 (22.2)
Asian	8 (1.8)
Other	9 (1.9)
Missing, n	29
**Tumor Histologic Type**, n(%)	
Cystic, mucinous, serous neoplasms	129 (28.4)
Epithelial neoplasms	1 (0.2)
Adenomas and adenocarcinomas	324 (71.4)
**Prior Malignancy**, n(%)	
No	414 (91.2)
Yes	40 (8.8)

Table S2: Summary statistics for cervical cancer cohort (TCGA-CESC)

	**Full sample (N = 296)**
**Survival proportion**	0.75
**Survival time (months)**	
Mean (SD)	35.1 (37.5)
Median (Q1, Q3)	22.5 (13.6, 43.6)
**Age (years)**	
Mean (SD)	48.1 (13.8)
Median (Q1,Q3)	46 (38, 56)
**Race**, n(%)	
White	207 (69.9)
Black | African American	32 (10.8)
Asian	19 (6.4)
Other	9 (3.0)

Missing, n	29
**Primary Tumor Size (T)**, n(%)	
Small (≤ 2cm)	138 (46.6)
Medium (> 2cm but ≤ 5cm)	70 (23.6)
Large (> 5cm)	17 (5.7)
Locally advanced/invasive	10 (3.4)
Non-invasive (Tis)	1 (0.3)
Unknown, n	60
**Lymph Node Involvement (N)**, n(%)	
None (N0)	131 (44.3)
Low (N1)	57 (19.3)
Moderate (N2)	-
Extensive (N3)	-
Unknown, n	108
**Metastasis status (M)**, n(%)	
No distant metastasis (M0)	107 (36.1)
Distant metastasis present (M1)	11 (3.7)
Unknown, n	178
**Tumor Histologic Type**, n(%)	
Cystic, mucinous, serous neoplasms	17 (5.7)
Complex epithelial neoplasms	3 (1.0)
Adenomas and adenocarcinomas	30 (10.1)
Squamous cell neoplasms	246 (83.1)
**Prior Malignancy**, n(%)	
No	288 (97.3)
Yes	8 (2.7)

Table S3: Survival outcomes by cluster for TCGA-UCEC

	Cluster 1	Cluster 2	Cluster 3	Cluster 4
Sample size	116	95	122	121
Survival proportion	0.819	0.874	0.779	0.851
Survival time (months)				
Mean (SD)	31.8 (31.0)	38.0 (31.7)	31.4 (22.2)	32.7 (27.6)
Median (Q1, Q3)	22.5 (14.8, 37.2)	29.6 (15.1, 56.7)	27.1 (15.9, 42.5)	25.2 (15.9, 37.6)

Table S4: Survival outcomes by cluster for TCGA-CESC

	Cluster 1	Cluster 2	Cluster 3	Cluster 4
Sample size	60	75	96	65
Survival proportion	0.683	0.720	0.771	0.815
Survival time (months)				
Mean (SD)	33.4 (31.4)	33.5 (35.3)	35.4 (42.9)	38.1 (37.5)
Median (Q1, Q3)	21.3 (15.6, 38.4)	25.9 (11.9, 40.8)	20.4 (12.1, 44.7)	24.5 (15.2, 47.8)

Table S5: Mean Area Under the Receiver Operating Characteristic Curve (AUC) for each classifier evaluated within each STRIVE-derived cluster for TCGA-UCEC. Performance was evaluated on a held-out test set following a single stratified 70/30 train/test split. Bold values indicate the highest performing classifier within each cluster.

Models	Cluster 1	Cluster 2	Cluster 3	Cluster 4
XGBoost	**0.672**	0.530	0.522	0.575
Gradient Boosting	0.489	**0.535**	0.392	0.352
Random Forest	0.626	0.230	0.474	**0.645**
Logistic Regression	0.431	0.350	0.425	**0.575**

Table S6: Mean Area Under the Receiver Operating Characteristic Curve (AUC) for each classifier evaluated within each STRIVE-derived cluster for TCGA-CESC. Performance was evaluated on a held-out test set following a single stratified 70/30 train/test split. Bold values indicate the highest performing classifier within each cluster.

Models	Cluster 1	Cluster 2	Cluster 3	Cluster 4
XGBoost	0.528	0.304	0.338	**0.594**
Gradient Boosting	0.347	**0.382**	0.250	0.320
Random Forest	0.431	0.176	0.279	**0.609**
Logistic Regression	**0.597**	0.549	0.487	0.359

Table S7. Comparison of latent-space clustering quality and stability across multimodal integration and single-modality models on TCGA-BRCA at k=6. STRIVE was compared with an otherwise matched VAE without attention, simple early concatenation, MOFA, a gene-expression-only attention-based VAE (GE-only), and a DNA-methylation-only attention-based VAE (DNAm-only). Clustering was performed using k-means on each model's latent representation. Values represent the mean and 95% confidence interval obtained by resampling 80% of patients without replacement over 1,000 iterations and re-clustering each subsample; consensus scores were obtained using an independent consensus-clustering procedure. Arrows indicate the favorable direction (↑ higher is better; ↓ lower is better). Minimum cluster size is reported as a diagnostic for potentially degenerate solutions.

Metric	STRIVE	VAE (no attention)	Simple concatenation	MOFA	GE-only VAE	DNAm-only VAE
**Silhouette score (↑)**	**0.2376** [0.2293, 0.2450]	0.2376 [0.2296, 0.2447]	0.2350 [0.2268, 0.2430]	0.1704 [0.1446, 0.1970]	0.2051 [0.1951, 0.2160]	0.2191 [0.2092, 0.2290]

**Davies–Bouldin index (↓)**	**1.1140** [1.0870, 1.1500]	1.1150 [1.0870, 1.1520]	1.1220 [1.0940, 1.1580]	1.4380 [1.3270, 1.5690]	1.2834 [1.233, 1.344]	1.2022 [1.1612, 1.2551]
**Calinski–Harabasz index (↑)**	**209.5** [203.3, 216.2]	209.7 [203.7, 216.3]	208.1 [201.6, 214.5]	137.6 [109.2, 156.5]	174.2 [166.5, 183.4]	192.3 [184.4, 201.2]
**Bootstrap ARI (↑)**	**0.8347** [0.5487, 0.9623]	0.8191 [0.5649, 0.9450]	0.7915 [0.4715, 0.9424]	0.4123 [0.2048, 0.9134]	0.673 [0.392, 0.881]	0.745 [0.461, 0.914]
**Consensus score, 1−PAC (↑)**	**0.8594** [0.8441, 0.8738]	0.8639 [0.8441, 0.8823]	0.8326 [0.8130, 0.8505]	0.5283 [0.4986, 0.5541]	0.7441 [0.7123, 0.7722]	0.7922 [0.7615, 0.8214]
**Minimum cluster size**	102.3 [91, 110]	103.2 [93, 110]	100.1 [89, 109]	6.6 [5, 8]	72.7 [61, 83]	84.1 [73, 94]

Supplementary Figures

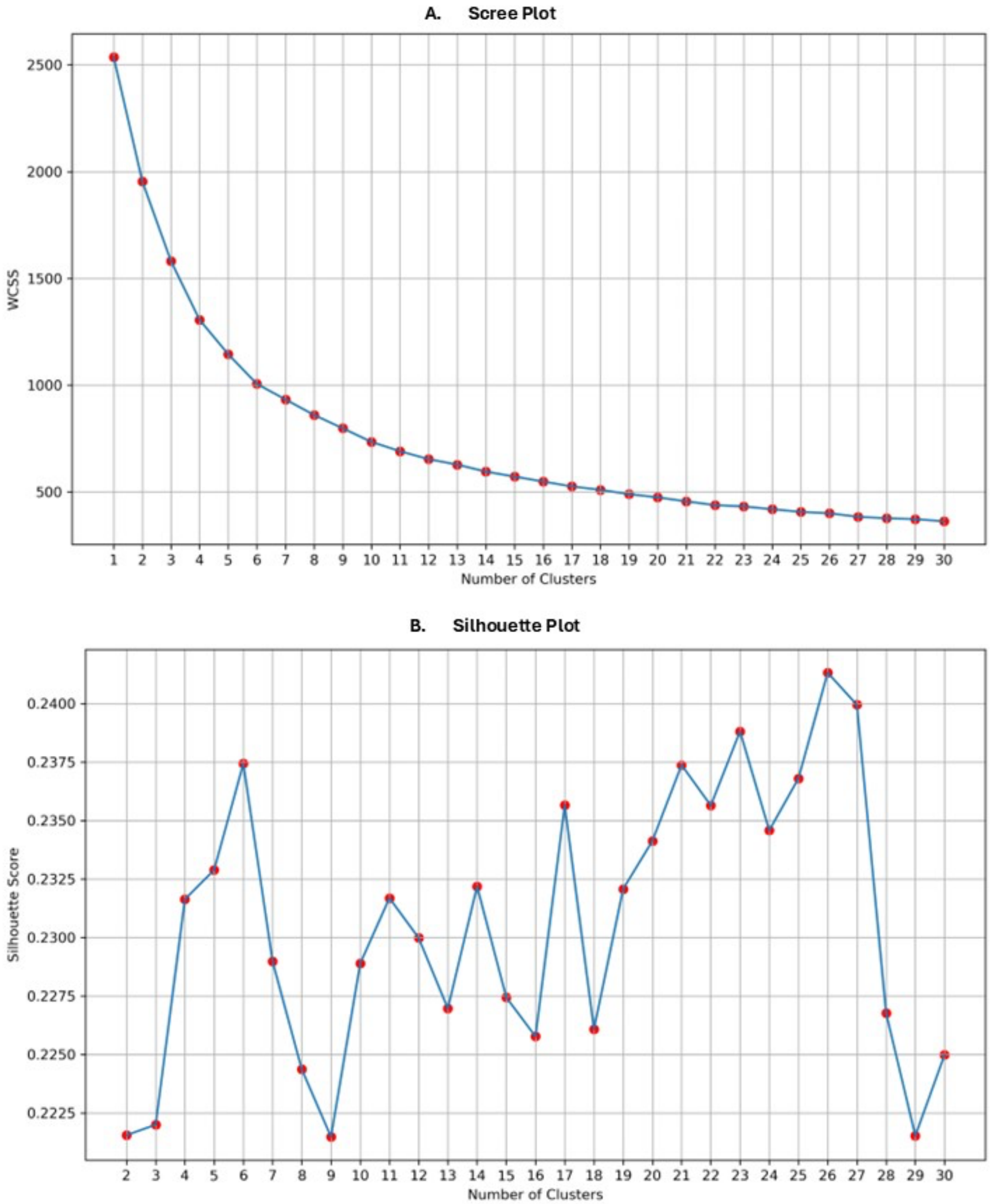

Figure S1. Internal cluster validation metrics for establishing the optimal number of clusters for TCGA-BRCA

(A) Within-cluster sum of squares (WCSS) plotted against the number of clusters to evaluate the rate of variance reduction. **(B)** Average silhouette width across varying cluster numbers to assess partition distinctness. The selection of four clusters was justified by both scree and silhouette evaluations, which revealed a distinct inflection point in explained variance alongside a local maximum for the silhouette coefficient.

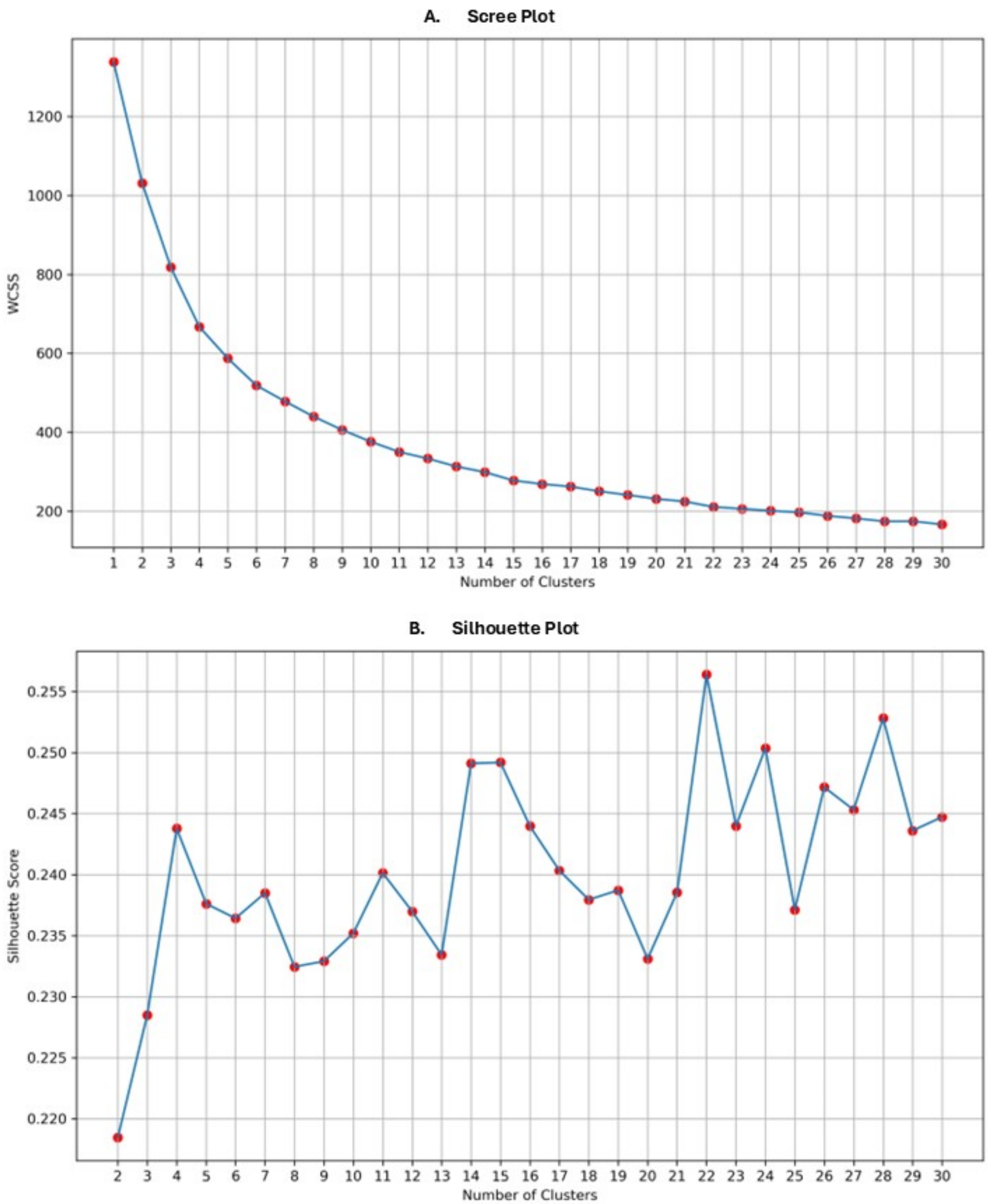

Figure S2. Internal cluster validation metrics for establishing the optimal number of clusters for TCGA-UCEC

(A) Within-cluster sum of squares (WCSS) plotted against the number of clusters to evaluate the rate of variance reduction. **(B)** Average silhouette width across varying cluster numbers to assess partition distinctness. The selection of four clusters was justified by both scree and silhouette evaluations, which revealed a distinct inflection point in explained variance alongside a local maximum for the silhouette coefficient.

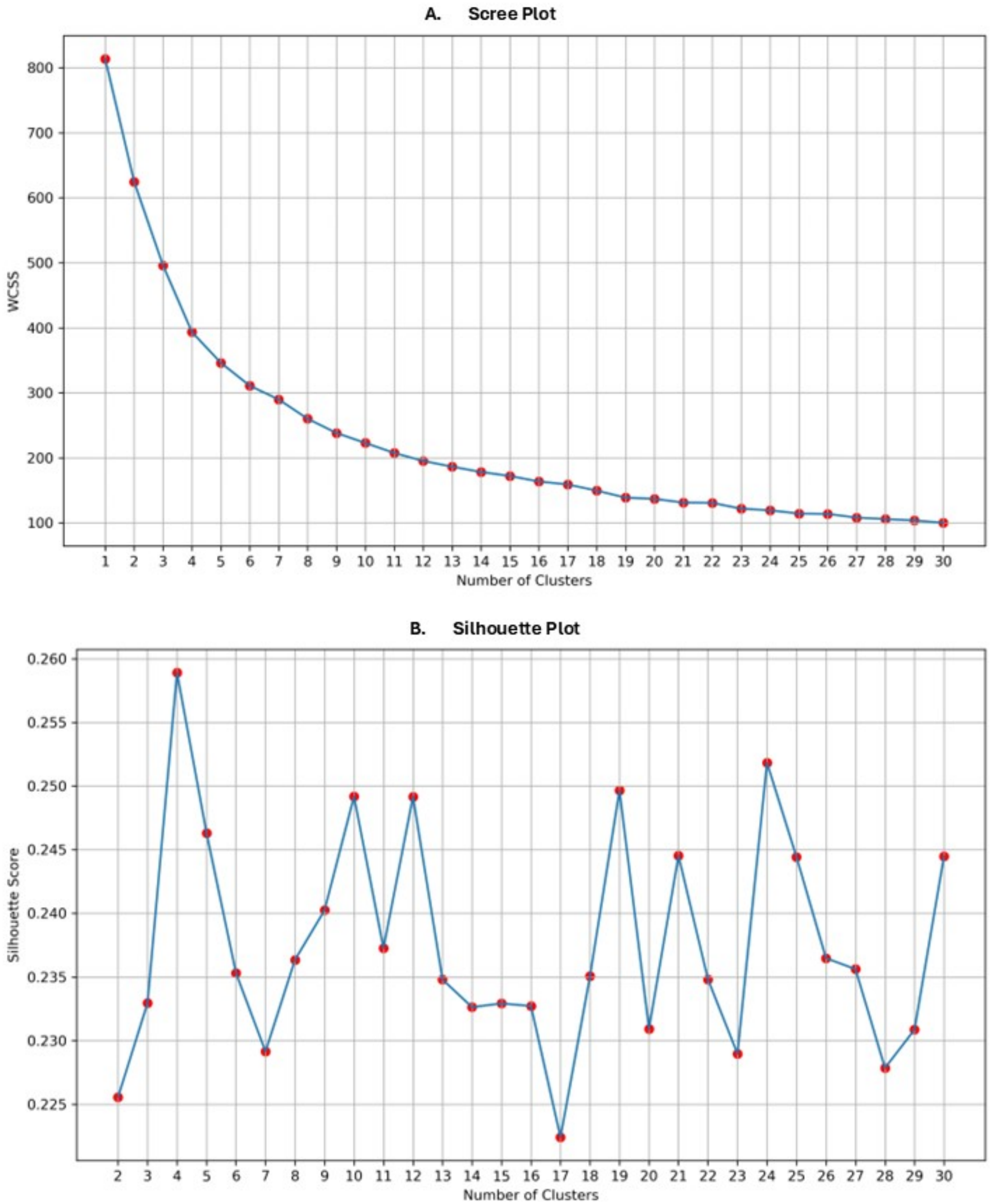

Figure S3. Internal cluster validation metrics for establishing the optimal number of clusters for TCGA-CESC

(A) Within-cluster sum of squares (WCSS) plotted against the number of clusters to evaluate the rate of variance reduction. **(B)** Average silhouette width across varying cluster numbers to assess partition distinctness. The selection of four clusters was justified by both scree and silhouette evaluations, which revealed a distinct inflection point in explained variance alongside a local maximum for the silhouette coefficient.

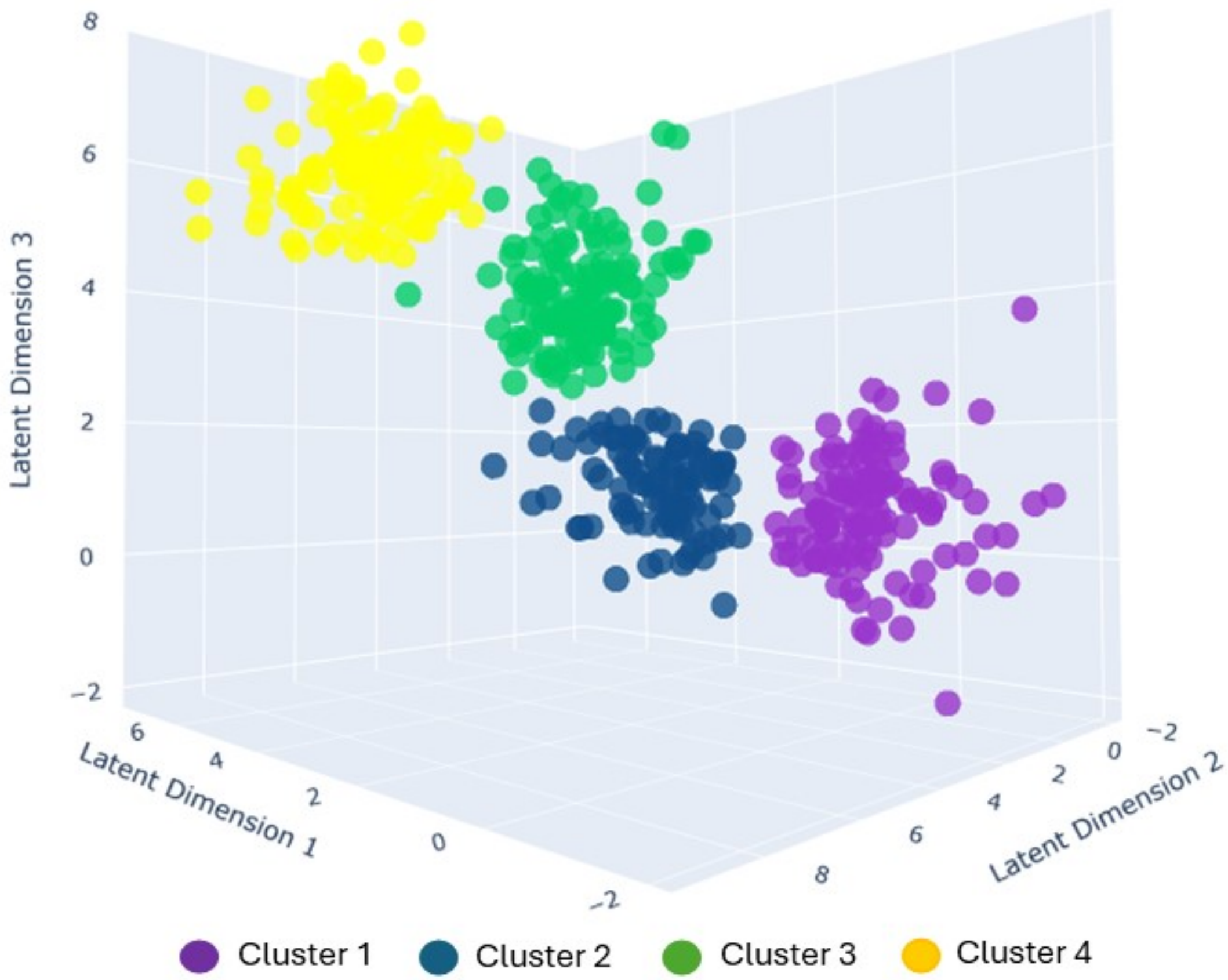

Figure S4. K-Means Clustering of Integrated Molecular Features using STRIVE-Guided Latent Embeddings for TCGA-UCEC

K-means clustering was applied to the learned latent embedding space, identifying four patient clusters represented in a 3D visualization. Each point corresponds to an individual patient, with colors indicating cluster assignment. Clusters include: Cluster 1 (purple, n = 116), Cluster 2 (blue, n = 95), Cluster 3 (green, n = 122), and Cluster 4 (yellow, n = 121).

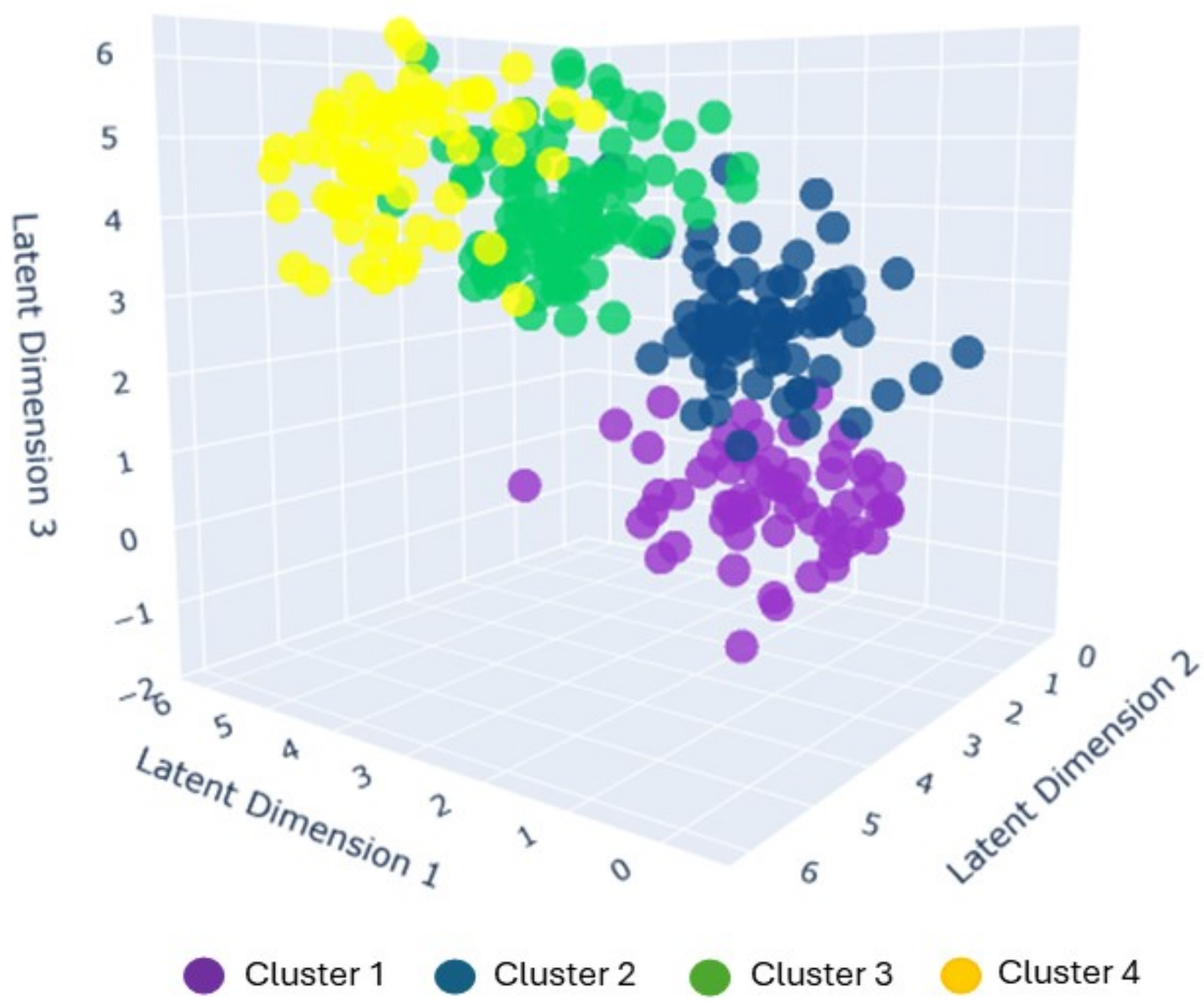

Figure S5. K-Means Clustering of Integrated Molecular Features using STRIVE-Guided Latent Embeddings for TCGA-CESC

K-means clustering was applied to the learned latent embedding space, identifying four patient clusters represented in a 3D visualization. Each point corresponds to an individual patient, with colors indicating cluster assignment. Clusters include: Cluster 1 (purple, n = 60), Cluster 2 (blue, n = 75), Cluster 3 (green, n = 96), and Cluster 4 (yellow, n = 65).

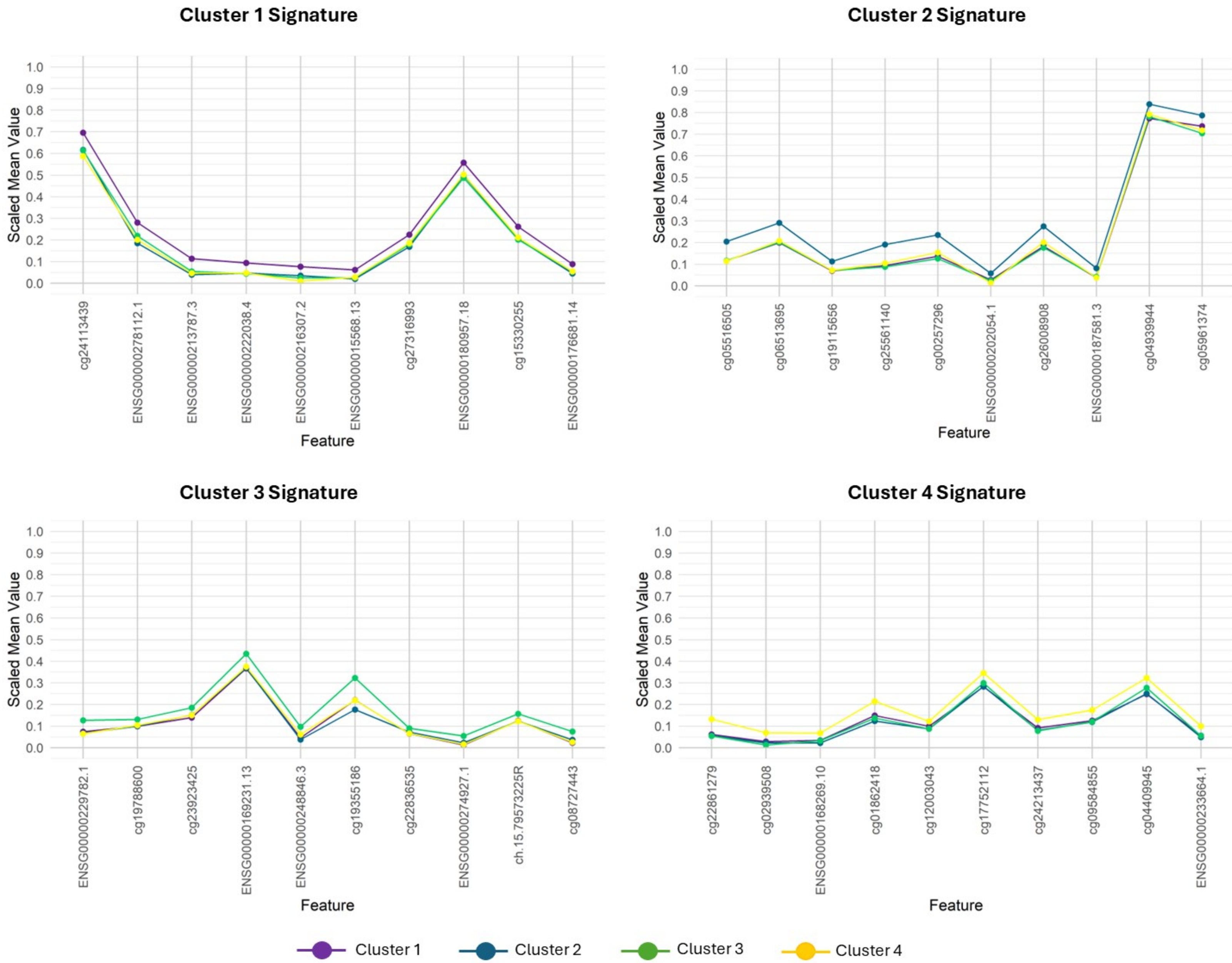

Figure S6. Integrated analysis of patient clustering and gene expression and DNA feature signatures for TCGA-UCEC

Molecular signature profiles for each of the four STRIVE-derived clusters, identified using standardized mean difference (SMD) analysis ($q < 0.05$) across gene expression and DNA methylation domains. Features were retained as cluster-specific markers only if consistently elevated in the target cluster relative to all others. DNA methylation probes are identified by CpG identifiers and gene expression features by gene symbols. Top driving features for three of the four clusters are from the DNA methylation domain (LDLR: cg24113439 for cluster 1; TNRC18: cg04939944 for cluster 2; cg1775212 and PSMB3:cg04409945 for cluster 4). In contrast, the leading feature for Cluster 3 belongs to the gene expression domain (*THBS3*: ENSG00000169231.13).

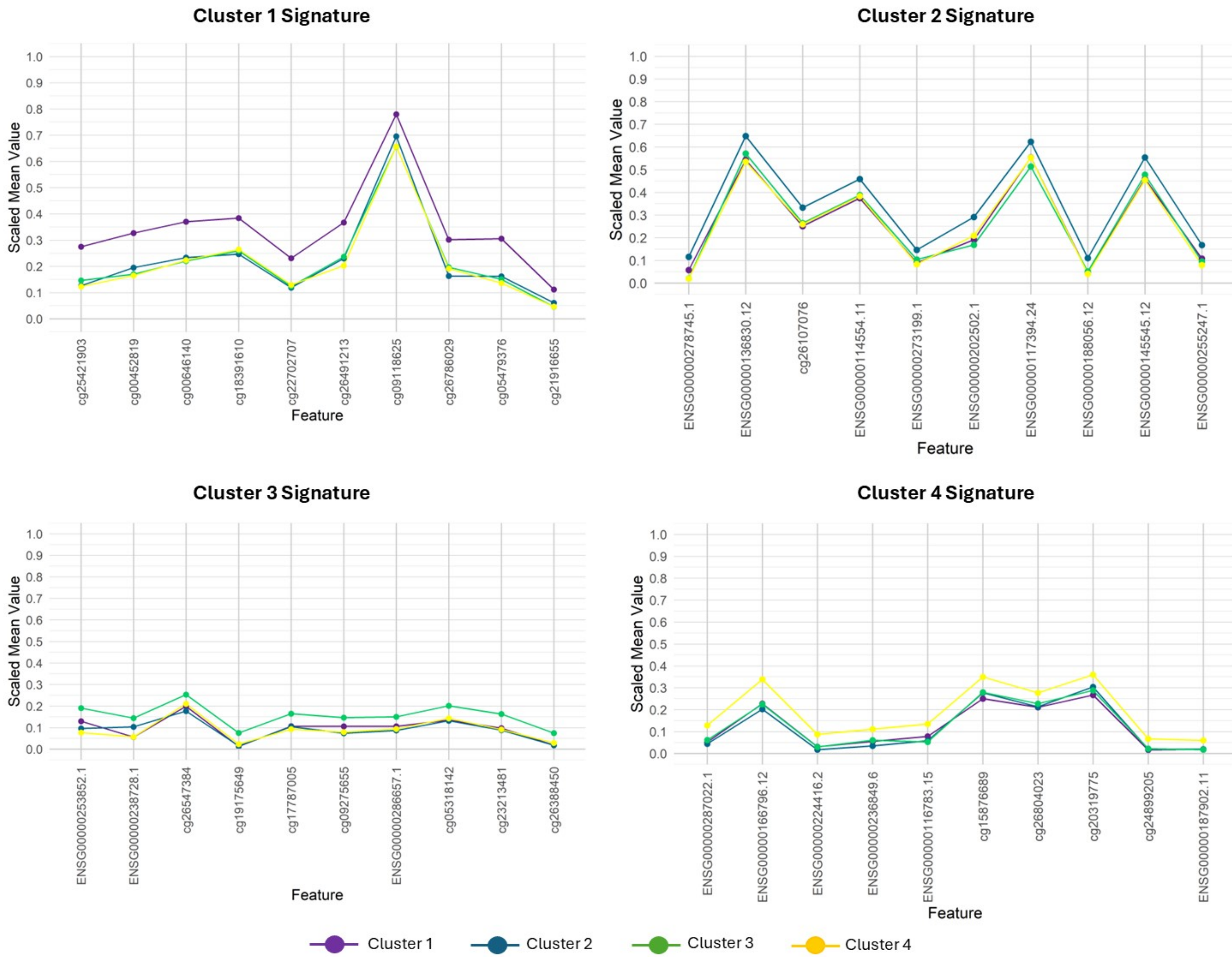

Figure S7. Integrated analysis of patient clustering and gene expression and DNA feature signatures for TCGA-CESC

Molecular signature profiles for each of the four STRIVE-derived clusters, identified using standardized mean difference (SMD) analysis ($q < 0.05$) across gene expression and DNA methylation domains. Features were retained as cluster-specific markers only if consistently elevated in the target cluster relative to all others. DNA methylation probes are identified by CpG identifiers and gene expression features by gene symbols. While the molecular signature for Cluster 1 is comprised exclusively of DNA methylation features, the remaining three clusters exhibit a multi-omic blend of both gene expression and DNA methylation features, with cluster 3 showing comparatively homogenous mean feature expression values.
